# Mid- and Long-Term Results of Covered Stents for Iatrogenic Common Femoral Artery Injury

**DOI:** 10.3390/biomedicines13123075

**Published:** 2025-12-12

**Authors:** Francesca Miceli, Giulia Demirxhiu, Alessia Di Girolamo, Antonio Marzano, Andrea Molinari, Rocco Cangiano, Marta Ascione, Francesco Ajmone, Gennaro Sardella, Massimo Mancone, Luca di Marzo, Wassim Mansour

**Affiliations:** 1 Vascular and Endovascular Surgery Division, Department of General Surgery, Specialties and Anaestesiology, Policlinico Umberto I, “Sapienza” University of Rome, Viale del Policlinico 155, 00161 Rome, Italy; francesca.miceli@uniroma1.it (F.M.); giulia.demirxhiu@uniroma1.it (G.D.); alessia.digirolamo@uniroma1.it (A.D.G.); antonio.marzano@uniroma1.it (A.M.); andrea.molinari@uniroma1.it (A.M.); rocco.cangiano@uniroma1.it (R.C.); marta.ascione@uniroma1.it (M.A.); luca.dimarzo@uniroma1.it (L.d.M.); 2 Cardiac Surgery Division, Department of Cardio-Thoraco-Vascular Surgery, Policlinico Umberto I, “Sapienza” University of Rome, Viale del Policlinico 155, 00161 Rome, Italy; francesco.ajmone@uniroma1.it (F.A.); gennaro.sardella@uniroma1.it (G.S.); massimo.mancone@uniroma1.it (M.M.)

**Keywords:** peripheral artery injury, covered-stent, vascular trauma, access-site complications, pseudoaneurysm, retrograde dissection, arterial perforation, arteriovenous fistula

## Abstract

**Background/Objectives:** The increasing use of endovascular procedures with common femoral artery (CFA) access has led to a rise in iatrogenic arterial injuries at this site. The most frequent injuries are pseudoaneurysms (PSA), retrograde dissections (RD), arteriovenous fistulas (AVF), and arterial perforations. Surgical repair is the standard treatment; however, the use of covered stents (CS) may represent a valid alternative, despite current instructions for use (IFU) not recommending CFA implantation. **Methods**: We conducted a single-center retrospective study on a prospectively maintained database. Patients undergoing transcatheter aortic valve repair (TAVR), endovascular aortic repair EVAR, diagnostic or therapeutic coronary angiography, or peripheral percutaneous transluminal angioplasty, who were subsequently treated for CFA injury with CS implantation between February 2015 and May 2024, were included. Endpoints were technical success (complete arterial repair), 30-day mortality, overall mortality, reintervention rates, and long-term stent patency. **Results**: A total of 41 patients were included: 10 (24.4%) PSA, 3 (7.3%) AVF, 27 (65.8%) perforations, and 2 (4.9%) RD. Of which 28 (68.3%) were treated with self-expandable CS and 13 (31.7%) with balloon-expandable CS. Additionally, 33 (80.5%) underwent urgent treatment. Technical success was achieved in 97.5%. Thirty-day mortality was 7.3%, with no procedure-related deaths. At a mean follow-up of 50.8 months (range 1–109), survival was 63.4%, with 100% stent patency and no procedure-related reinterventions. **Conclusions**: CS implantation for CFA iatrogenic injuries achieved high technical success and excellent long-term patency, representing a viable alternative to open repair. Further studies are needed to integrate CS use for CFA injuries into treatment algorithms and to update device IFUs accordingly.

## 1. Introduction

During the last decades, the prevalence of iatrogenic vascular injuries has increased as more percutaneous arterial catheterizations are used to manage several cardiovascular diseases [1]. As well reported in the literature, the common femoral artery (CFA) is the predominant site used as a large-bore puncture site. However, recently the axillary and subclavian artery have also become a dominant access site, especially during complex endovascular aortic procedures [2].

Many different vascular closure devices are available to close the access site at the end of the procedure. Nevertheless, complications related to vascular access are quite frequent, reaching up to 20% following transcatheter aortic valve repair (TAVR) and endovascular aortic repair (EVAR) [3,4,5]. The overall incidence reported from studies on large case series following therapeutic and diagnostic procedure is between 0.2 and 1% [6]. A sub-analysis of injuries based on the procedure type (diagnostic versus therapeutic) reveals a higher incidence of injury following therapeutic procedures than for diagnostic procedures, with a rate of 3% and 1%, respectively [7].

The most frequent access site related injuries are pseudoaneurysm (PSA), retrograde dissection (RD), arterial perforation and arteriovenous fistula (AVF) [8].

For a long time the first-choice treatment for iatrogenic peripheral arterial injury was the open surgical repair with a high technical success rate. However, this approach is frequently associated with complications such as hematoma, lymphocele, and impaired wound healing, which may predispose the patient to surgical site infection. In the daily clinical practice, the burden of arterial iatrogenic injuries in an urgent setting, and in patients with several comorbidities, seriously challenges this option.

Nowadays, endovascular techniques with implantation of covered stent (CS) have been proposed as alternative to open surgical repair in those cases affected by CFA injury, not only in an urgent setting but also in an elective regimen, due to the possibility of being performed under local anesthesia with a less invasive approach and mainly indicated in patients with several comorbidities or in those unfit for open surgery.

The recent literature supports the efficacy and durability of this approach [1,9,10]. One of the largest published studies included 136 patients who underwent endovascular repair with 30 Viabahn and 117 VBX stent-grafts (W.L.Gore) at the level of the proximal CFA and distal external iliac artery. Indications for intervention were active bleeding in 92 patients (68%), flow-limiting dissection in 41 patients (30%), and symptomatic arteriovenous fistula in 3 patients (2%). The study reported a primary technical success rate of 100%. During a 3-year follow-up, stent-grafts patency remained 100% in all but one case, in which target lesion revascularization was required due to neointimal hyperplasia. No stent rupture, stenosis, or kinking was observed, supporting the safety and durability of these devices in the treatment of proximal CFA and distal external iliac artery injuries [11].

Nevertheless, systematic indications to use CS in this setting still represent a scientific gap, since the instructions for use of these devices do not include their use in peripheral artery injury, especially at the level of the common femoral artery.

Therefore, the present study analyzed procedural and clinical outcomes in a cohort of patients affected by iatrogenic peripheral artery injury at the CFA level and treated with CS implantation with a mid- and long-term follow-up time.

## 2. Materials and Methods

A retrospective study was conducted on a prospectively compiled computerized database on patients presenting a common femoral artery (CFA) iatrogenic arterial injury treated by endovascular repair with covered stent-grafts (CS) from February 2015 to May 2024 in an Italian tertiary university center.

Index procedures were as follows: transcatheter aortic valve repair (TAVR), diagnostic or therapeutic coronarography, endovascular aortic repair (EVAR) procedures, diagnostic angiography, or percutaneous trans-arterial angioplasty. We included all patients who had undergone a diagnostic or therapeutic percutaneous procedure performed by interventional cardiologists, interventional radiologists and vascular surgeons, and afterwards developed an iatrogenic injury of the CFA.

The inclusion criteria were as follows: (1) major vascular access site complications such as pseudoaneurysms (PSA), arteriovenous fistulas (AVF), and retrograde dissection (RD); (2) failure of closure device systems leading to active bleeding; (3) careful evaluation by a multidisciplinary team composed of interventional radiologists, interventional cardiologists, and vascular surgeons.

The clinical diagnosis of the iatrogenic injury was confirmed by intra-operative angiography, Doppler ultrasound (DUS), and/or CT angiography (CTA).

Patients without a clear active bleeding site or in absence of acute limb ischemia were treated in an elective setting. Nevertheless, those patients with unstable hemodynamic status and active bleeding or any sign of acute limb ischemia were treated in an urgent setting.

Three types of stents available at our center were used (GORE^®^ VIABAHN^®^ VBX Balloon-Expandable Endoprosthesis, Gore Inc., Flagstaff, AZ, USA; GORE^®^ VIABAHN^®^ Self-Expandible Endoprosthesis with PROPATEN Bioactive Surface, Gore Inc., Flagstaff, Arizona, or Advanta V12 Balloon-Expandable Covered Stent, Atrium Medical Corporation, Hudson, NH, USA). The choice of the stent-graft was based on the diameter of the vessel and location and length of the iatrogenic lesion. All the procedures were performed by a contralateral cross-over percutaneous approach by placing a covered stent-graft (CS) at the level of the lesion and when not feasible, for instance in those cases treated by EVAR, an ipsilateral superficial femoral artery or an upper extremity access were performed.

At the end of the procedure, an angiography was performed to assure the hemostasis and the correct position of the stent-graft. A closure device system was used to close the percutaneous access site.

All patients with high cardiovascular and non-cardiovascular comorbidities were held in the Intensive care Unit for at least 24 h after the procedure. Each patient underwent a clinical examination and a DUS evaluation at first post-operative day, then a DUS examination at 30 days, at 6 months, at 12 months, and then annually.

The primary endpoint of the study was the technical success in terms of a complete arterial repair, restoration of a regular blood flow, the patency of the stent-graft and the run-off vessels. The secondary endpoints were 30-day mortality, cumulative mortality, and the incidence of complications such as restenosis, kinking, occlusion, infection [12], rupture of the stent-graft, open conversion and the rate of reinterventions, and major or minor amputations.

### 2.1. Statistical Analysis

Patient’s data were reported in an Excel sheet (Microsoft Inc., Redmond, WA, USA).

Continuous variables are given as mean and standard deviation. Categorical variables are given as number (percentage). A *p* value of <0.05 was considered statistically significant. All analyses were calculated using SPSS version 26 (IBM Corp., Armonk, NY, USA). Long-term survival and freedom from reintervention were determined by life-table analysis, Kaplan–Meier curves, and log-rank tests.

### 2.2. Ethical Requirements

This study complied with the principles of the Declaration of Helsinki. Informed consent of the patients was obtained for the procedures but was not required for the study.

Ethical approval was waived due to the retrospective nature of the study, according to our Ethical Committee.

## 3. Results

Between February 2015 and May 2024, a total of 41 patients underwent endovascular repair for iatrogenic injury of CFA following endovascular procedure such as transcatheter aortic valve repair (TAVR), diagnostic or therapeutic coronarography, endovascular aortic repair (EVAR) procedures, diagnostic angiography, or percutaneous trans-arterial angioplasty (PTA).

The patients’ characteristics and the main cardiovascular (CV) risk factors are reported in Table 1.

Analyzing the index procedures, 22 patients underwent TAVR, 3 patients a therapeutic coronarography, 2 patients a diagnostic coronarography, 4 patients a therapeutic PTA, and 10 patients underwent EVAR procedures.

**Figure 1:**
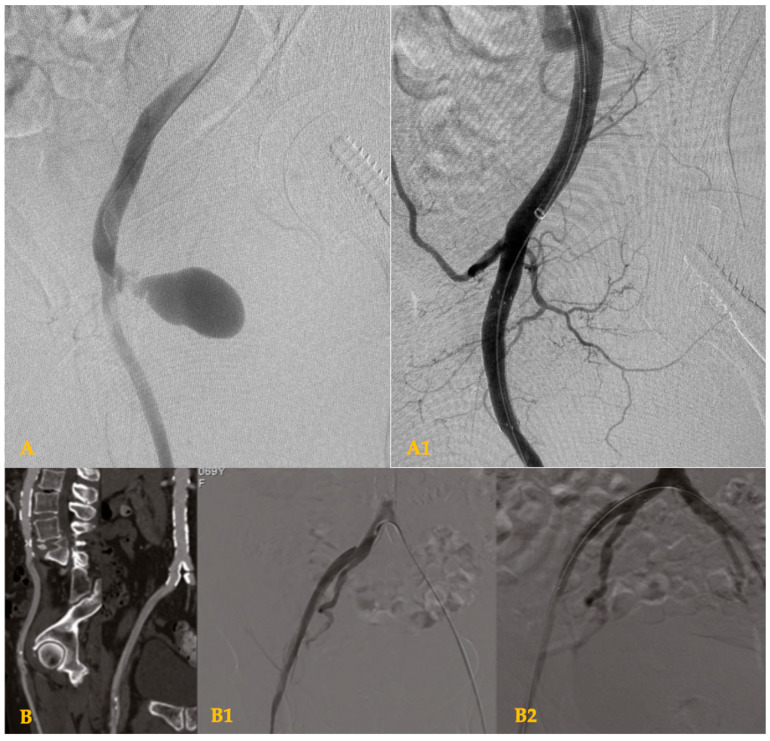
Angiographic images of iatrogenic common femoral artery injuries. (**A**) Pseudoaneurysm of the common femoral artery, treated with covered stent at this level (**A1**). (**B**) Retrograde dissection of the iliac-femoral axis, treated with two covered stents through a cross-over technique (**B1**,**B2**).

According to the lesion type, 10 (24.4%) patients developed a PSA, 2 (4.9%) AVF, 27 (65.8%) arterial perforations, and 2 (4.9%) RDs (Table 2) (Figure 1).

Regarding the operative setting, 33 (80.5%) patients were treated in urgency because of active bleeding at the vascular access site with unstable hemodynamic conditions. The remaining 8 patients (19.5%) were treated, after a first attempt of resolution through a manual compression, in an elective setting because of absence of clinical symptomatology.

Concerning the type of anesthesia, 39 patients (95.1%) were treated under local anesthesia, whereas the remaining 2 cases were treated in emergency setting during another procedure which already required a general anesthesia.

An accurate evaluation of the vessel’s characteristics and procedural characteristics are reported in Table 3.

Notably, in three patients, two overlapped Gore Viabahn stent-graft at the level of the CFA were necessary because the length of the lesion was too extensive and only one stent-graft was not sufficient to effectively exclude the target lesion. In one case, coverage of the ostium of the deep femoral artery occurred, without any clinical sequelae during the follow-up.

At the end of each procedure, all patients underwent a final angiography to evaluate the proper position and the patency of the stent-graft, a regular blood flow through the stent and on the run-off vessels, and the complete exclusion of the target lesion.

Technical success rate was achieved in 40 (97.5%) patients. One patient died from massive bleeding, due to CFA perforation following a TAVR procedure. Firstly, two Viabahn CS were implanted at the lesion level in an urgent setting, with insufficient bleeding control requiring an open conversion. Nevertheless, this patient required massive transfusion and died a few hours after surgery.

In one case, the patient was affected by CFA pseudoaneurysm complicated by skin fistulization and evacuation of the hematoma via surgical incision was performed as an adjunctive procedure to the endovascular repair.

### 3.1. 30-Day Follow-Up

The 30-day mortality rate was 7.3%. Notably, three patients died at 30 days. All patients were treated as index procedure with TAVR. One patient died within 24 h from heart failure, the second at 28 days from acute respiratory failure, and the third at 30 days from acute myocardial infarction (AMI).

### 3.2. 1-Year Follow-Up

We reported a 10.81% mortality rate at 1-year of follow-up: one patient died from SarS-CoV-2 pulmonary infection three months after the index-procedure, one patient died from aorto-enteric fistula after 7 months from the EVAR; one patient died due to end-stage myeloid leukemia nine months after; and the last patient died due to AMI ten months later.

No evidence of procedural-related death was reported and the DUS examination at 1 year showed the patency of the stent-graft in absence of any complications such as intimal hyperplasia or restenosis and stent-graft rupture or migration.

No major or minor amputation were reported.

### 3.3. Long-Term Follow-Up

**Figure 2:**
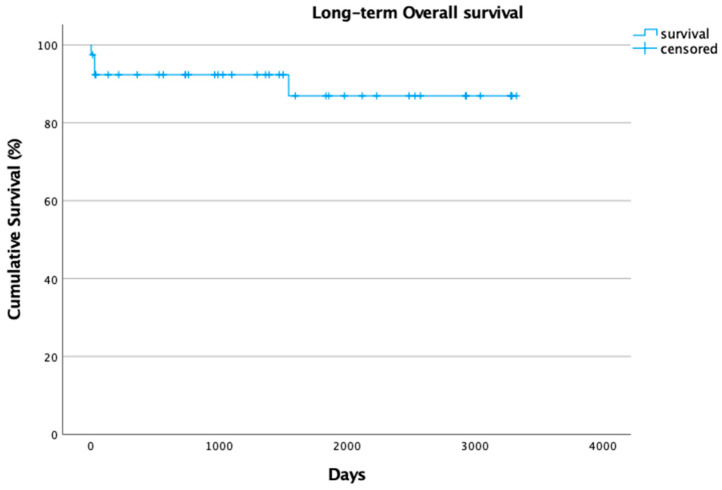
Kaplan–Meier curve shows long-term cumulative survival rate (%).

Of the remaining 33 patients, at mean follow-up time of 54 months (Range: 1–108 months), 26 (63,4%) patients showed good clinical conditions, a stent patency of 100% in absence of any procedural-related reinterventions. Nevertheless, of the 33 patients, 7 patients were lost at long-term follow-up (Figure 2).

## 4. Discussion

Traditional open surgery for peripheral arterial injury due to access site complications are known to be associated with increased mortality and morbidity, especially in an urgent setting [13].

As reported in the literature the main arterial percutaneous access site for diagnostic or therapeutic procedures is the common femoral artery (CFA) [2]. Nevertheless, the risk of femoral artery injury associated with large-bore devices is not negligible [3]. Although open surgery has a high technical success rate, the 30-day mortality rate reaches up to 14% [14]. Therefore, an off-label endovascular percutaneous procedure with CS implantation is a viable alternative in those patients with high-risk comorbidities and at high risk for surgical repair, also at the level of the CFA.

To this date, CS implantation at the level of the CFA is conventionally contraindicated, because the artery is under constant bending, torsion, and external compression by the inguinal ligament that could lead to stent kinking, fracture, or occlusion [15].

In a recent published study, 136 individuals were treated with 30 Viabahn and 117 Viabahn VBX (W.L. Gore) stent-grafts at the level of the proximal common femoral artery and the distal external iliac artery. Intervention was indicated for 92 patients (68%) with bleeding, 41 patients (30%) with flow-limiting dissection, and 3 patients (2%) with symptomatic AVF. The primary technical success rate was 100%. Apart from one patient who had target lesion revascularization because of neointimal hyperplasia, a limited 3-year follow-up (101/136 patients) revealed 100% patency with no signs of stent breakage, stenosis, or kinking [11].

We reported 41 iatrogenic injuries at the level of the CFA treated with implantation of a CS with a technical success rate of 97.6%. Only one patient died intraoperatively for massive bleeding, due to CFA perforation following a TAVR procedure after a first endovascular attempt of resolution. At 30-day follow-up the rate of reinterventions was 0%, and at an average follow-up time of 50.8 months, the rate of amputation and reintervention was 0%, with a stent patency rate of 100%.

In our series, in only one case was a bailout surgery necessary. The patient presented CFA perforation following TAVR bleeding. Firstly, two Viabahn CS were implanted at the lesion level in an urgent setting. Then, an open conversion for uncontrolled bleeding was necessary but with failure.

Maurina et al. reported that two cases required bailout surgery, but in 96.2% of cases, covered stents were effectively implanted to address vascular problems following TAVR. In-hospital death occurred for one patient. Before the discharge no instances of fracture or in-stent occlusion were found using Doppler ultrasound. Only one patient had new-onset claudication, and no occurrences of symptomatic limb ischemia were observed during a median follow-up of 429 days [16].

Nevertheless, recent case reports and retrospective studies showed good mid-term results in terms of stent-graft patency and rate of reinterventions after endovascular treatment with implantation of a CS at the level of the CFA [13].

Self-expandable stent-grafts offer superior flexibility, making them particularly suitable for tortuous arterial segments, and have demonstrated favorable outcomes in managing iatrogenic injuries across both peripheral and visceral vessels. Balloon-expandable stent-grafts, although typically associated with a slightly larger delivery profile, represent a valid alternative—especially in peripheral arteries—because they allow precise deployment and accurate sizing to the injured vessel. In a study by Ruffino et al., the use of both self-expandable and balloon-expandable stent-grafts resulted in excellent technical success (100%) and high clinical success (88%), further supporting the effectiveness of endovascular repair in this context [13]. Our results, compared to the literature, showed a 97.6% technical success without any sensitivity or motricity deficiency. Concerning the in-hospital mortality rate, Kufner et al. reported 10% in-hospital mortality rate, while our cohort of patients was characterized by a 7.3% in-hospital mortality: mostly occurring in elderly patients with ASA IV classification, treated in an urgent setting [17]. The overall mortality rate at one year follow-up in our study is 10.81%, while Kufner et al. reported a 20% mortality rate; this may be related to demographical and comorbidity differences between the two cohorts.

In a recent multicenter retrospective study enrolling 23 patients treated with balloon-expandable covered stents for vascular access site complications, at an average follow-up time of 18 months, only one patient underwent reintervention due to stent-graft occlusion [9].

Although the small sample of patients and the retrospective nature of our study provide some limitations of the study, the principal strength of our study lies in its long-term follow-up (mean 50.8 months, range 1–109 months), during which we observed 100% stent patency and no local or systemic complications, including amputations or procedure-related reinterventions. These results, consistent with the existing literature, support the role of CS implantation as a safe and durable alternative to surgery for iatrogenic arterial injuries. Revision of current CS instructions for use is warranted to incorporate their implantation into treatment algorithms for iatrogenic common femoral artery injuries.

## 5. Conclusions

Based on our results, the endovascular treatment of iatrogenic arterial injuries using self-expandable and balloon-expandable stent-grafts is a safe and effective alternative strategy, with a 97.6% technical success rate and a high patency rate at 12 months and at long-term follow-up in absence of procedure-related complications and reinterventions.

Although our findings suggest CS implantation as a valid alternative to open surgery, a prospective randomized trial on a larger population could properly validate our results.

Moreover, further studies are needed to include CS implantation both in the treatment algorithm and to update the instructions for use of CS by including iatrogenic arterial injuries among the indications.

## Figures and Tables

**Table 1:** Patient’s characteristics and cardiovascular risk factors.

Risk Factors	N, %
Mean age (years)	79 (range, 48–92)
HTN	37 (90.2)
Diabetes Mellitus	11 (26.8)
Dyslipidemia	23 (56.1)
Smoking	20 (48.9)
Atrial Fibrillation	11 (26.8)
COPD	3 (7.3)
ESRD	16 (39)
AMI	11 (26.8)
CAD	17 (41.5)
PAD	9 (21.9)

COPD: chronic obstructive pulmonary disease; ESRD: end-stage renal disease; AMI: acute myocardial infarction; CAD: chronica cardiac disease; PAD: peripheral arterial disease.

**Table 2:** Index procedure and associated iatrogenic arterial injury.

Index Procedure	PSA (n)	AVF (n)	Perforation (n)	RD (n)
TAVR	6	-	16	-
Therapeutic coronarography	-	1	2	-
Diagnostic coronarography	-	-	2	-
PTA	-	1	1	2
EVAR	4	-	6	-

PSA: pseudoaneurysm; AVF: arteriovenous fistula; RD: retrograde dissection; TAVR: transcatheter aortic valve repair; PTA: percutaneous trans-arterial angioplasty; EVAR: endovascular aortic repair; “-“ none.

**Table 3:** Procedural characteristics.

Procedural Characteristics	N, (Range)
Mean proximal diameter of the CFA (mm)	8.2 (5.4–10.2)
Mean distal diameter of the CFA (mm)	8.4 (5.6–10.5)
Mean length of the lesion	25.4 (23.2–130.6)
Gore Viabahn	31
Gore VBX	4
Advanta V12	9
Mean length of the stent (mm)	48.2 (29–150)
Mean diameter of the stent (mm)	8.6 (6–11)
Mean duration of the procedure (min)	35 (10–135)
Mean fluoroscopy time (min)	9 (5–35)

CFA: common femoral artery.

## Data Availability

The original contributions presented in this study are included in the article. Further inquiries can be directed to the corresponding author.

## References

[1] Rizk T., Patel D., Dimitri N.G., Mansour K., Ramakrishnan V. (2020). Iatrogenic Arterial Perforation During Endovascular Interventions. Cureus.

[2] Mesnard T., Vacirca A., Baghbani-Oskouei A., Sulzer T.A.L., Savadi S., Kanamori L.R., Tenorio E.R., Mirza A., Saqib N., Mendes B.C. (2024). Prospective Evaluation of Upper Extremity Access and Total Transfemoral Approach during Fenestrated and Branched Endovascular Repair. J. Vasc. Surg..

[3] Heger T., Strauß S., Blessing E., Andrassy M., Erbel C., Müller O.J., Chorianopoulos E., Pleger S., Leuschner F., Korosoglou G. (2017). Short and Long-Term Results after Endovascular Management of Vascular Complications during Transfemoral Aortic Valve Implantation. Acta Cardiol..

[4] Cuozzo S., Martinelli O., Brizzi V., Miceli F., Flora F., Sbarigia E., Gattuso R. (2023). Early Experience with Ovation Alto Stent-Graft. Ann. Vasc. Surg..

[5] Martinelli O., Cuozzo S., Miceli F., Gattuso R., D’Andrea V., Sapienza P., Bellini M.I. (2023). Elective Endovascular Aneurysm Repair (EVAR) for the Treatment of Infrarenal Abdominal Aortic Aneurysms of 5.0–5.5 Cm: Differences between Men and Women. J. Clin. Med..

[6] Minici R., Paone S., Talarico M., Zappia L., Abdalla K., Petullà M., Laganà D. (2020). Percutaneous Treatment of Vascular Access-Site Complications: A Ten Years’ Experience in Two Centres. CVIR Endovasc..

[7] Tonnessen B.H. (2011). Iatrogenic Injury from Vascular Access and Endovascular Procedures. Perspect. Vasc. Surg. Endovasc. Ther..

[8] Rizzo A.N., Patel K., Perdue J., Henry J.C. (2024). Endovascular Covered Stent Graft Repair by Through-and-through Snaring of an Acute Traumatic Femoral–Femoral Arteriovenous Fistula Following a Gunshot Wound. J. Vasc. Surg. Cases Innov. Tech..

[9] Szentiványi A., Borzsák S., Süvegh A., Bérczi Á., Szűcsborús T., Ruzsa Z., Fontos G., Szalay C.I., Papp R., Molnár L. (2024). Midterm Outcome of Balloon-Expandable Covered Stenting of Femoral Access Site Complications. J. Clin. Med..

[10] Drouelle E., De Poli F., Couppie P., Uhry S., Heyer H., Morel O., Ohlmann P., Hess S., Kibler M., Leddet P. (2019). Suivi Clinique Après Implantation d’un Stent Couvert Dans l’artère Fémorale Commune Au Décours Immédiat d’une Complication Vasculaire per-TAVI. Ann. Cardiol. Angeiol..

[11] Wilderman M., Tateishi K., O’Connor D., Simonian S., Ratnathicam A., Cook K., De Gregorio L., Hmoud H., De Gregorio J. (2024). Safety and Efficacy of Covered Stent Grafts in the Treatment of Emergent Access Related Complications. Cardiovasc. Revascularization Med..

[12] Ascione M., Dajci A., Cangiano R., Marzano A., Molinari A., Miceli F., Di Girolamo A., Leanza C., Oliva A., Di Marzo L. (2024). Open Surgical Conversion of Popliteal Endograft Infection: Case Reports and Literature Review. Biomedicines.

[13] Ruffino M.A., Fronda M., Varello S., Discalzi A., Mancini A., Muratore P., Rossato D., Bergamasco L., Righi D., Fonio P. (2020). Emergency Management of Iatrogenic Arterial Injuries with a Low-Profile Balloon-Expandable Stent-Graft. Medicine.

[14] Asmar S., Bible L., Obaid O., Tang A., Khurrum M., Castanon L., Ditillo M., Joseph B. (2021). Open vs Endovascular Treatment of Traumatic Peripheral Arterial Injury: Propensity Matched Analysis. J. Am. Coll. Surg..

[15] Troisi N., Bertagna G., Berchiolli R. (2024). RIvaroxaban and VAscular Surgery (RIVAS): Insights from a Multicenter, Worldwide Web-Based Survey. Int. Angiol..

[16] Maurina M., Condello F., Mangieri A., Sanz-Sanchez J., Stefanini G.G., Bongiovanni D., Cozzi O., Leone P.P., Baggio S., Gasparini G. (2022). Long Term Follow-up after Balloon Expandable Covered Stents Implantation for Management of Transcatheter Aortic Valve Replacement Related Vascular Access Complications. Catheter. Cardiovasc. Interv..

[17] Kufner S., Cassese S., Groha P., Byrne R.A., Schunkert H., Kastrati A., Ott I., Fusaro M. (2015). Covered Stents for Endovascular Repair of Iatrogenic Injuries of Iliac and Femoral Arteries. Cardiovasc. Revascularization Med..

